# Restoration of serum estradiol and reduced incidence of miscarriage in patients with low serum estradiol during pregnancy: a retrospective cohort study using a multifactorial protocol including DHEA

**DOI:** 10.3389/frph.2023.1321284

**Published:** 2024-01-04

**Authors:** Phil Boyle, Karolina Andralojc, Susanne van der Velden, Shahpar Najmabadi, Theun de Groot, Craig Turczynski, Joseph B. Stanford

**Affiliations:** ^1^ International Institute for Restorative Reproductive Medicine, London, United Kingdom; ^2^ NeoFertility Clinic, Dublin, Ireland; ^3^ Department of Medical Microbiology, Radboud University Medical Center, Nijmegen, Netherlands; ^4^ Kath. Karl-Leisner-Klinikum, Kleve, Germany; ^5^ Office of Cooperative Reproductive Health, Department of Family and Preventive Medicine, University of Utah, Salt Lake City, UT, United States; ^6^ Department of Medical Microbiology and Infectious Diseases, Canisius Wilhelmina Hospital, Nijmegen, Netherlands; ^7^ Billings Ovulation Method Association-USA, Saint Cloud, MN, United States

**Keywords:** pregnancy, miscarriage, estradiol, dehydroepiandrosterone, restorative reproductive medicine

## Abstract

**Background:**

Low serum estradiol in early pregnancy is associated with an elevated risk of miscarriage. We sought to determine whether efforts to restore low blood estradiol via estradiol or dehydroepiandrosterone (DHEA) supplementation would reduce the risk of miscarriage as part of a multifactorial symptom-based treatment protocol.

**Methods:**

This retrospective cohort study included women with low serum estradiol levels in early pregnancy, defined as ≤50% of reference levels by gestational age. Estradiol or DHEA were administered orally, and the primary outcome measure was serum estradiol level, in reference to gestational age. The secondary outcome measures included miscarriage, birth weight, and gestational age at birth.

**Results:**

We found no significant effect of estradiol supplementation on serum estradiol levels referenced to gestational age, while DHEA supplementation strongly increased estradiol levels. For pregnancies with low estradiol, the miscarriage rate in the non-supplemented group was 45.5%, while miscarriage rate in the estradiol and DHEA supplemented groups were 21.2% (*p* = 0.067) and 17.5% (*p* = 0.038), respectively. Birth weight, size, gestational age, and preterm deliveries were not significantly different. No sexual abnormalities were reported in children (*n* = 29) of DHEA-supplemented patients after 5–7 years follow-up.

**Conclusions:**

In conclusion, DHEA supplementation restored serum estradiol levels, and when included in the treatment protocol, there was a statistically significant reduction in miscarriage.

## Introduction

Human pregnancy is a complex process requiring tight regulation of the maternal system to support fetal development and maintain a successful pregnancy. Dysregulation of the maternal system or genetic abnormalities of the fetus may lead to pregnancy loss, most commonly as miscarriage (1). In some cases, the cause for miscarriage is found, which may be related to genetic, infectious or immunological factors, or result from abnormalities with implantation, uterine anatomy or the endocrine system (2, 3). Unfortunately, the etiology is not identified in most cases. This may be due to underlying pathologies not identified in routine clinical investigation or from factors not yet known to play a role in an adverse pregnancy outcome (2).

One factor may be low levels of the hormone estradiol, which was discovered almost a century ago (4). Estradiol plays an essential role in pregnancy, as demonstrated in animal models. In mice, estradiol was shown to be essential for implantation (5). In baboons, Albracht et al. demonstrated that a strong reduction in estradiol levels induced miscarriage in 50% of pregnancies (6). These miscarriages were fully prevented by the supplementation of estradiol (6). In humans, the importance of estradiol in pregnancy is supported by the fact that the aromatase inhibiting drug letrozole is currently under investigation as a cotreatment to induce abortion (7).

In human pregnancy, estradiol production increases ∼5 days after conception (8), via the corpus luteum and then around 9 weeks of gestation by the placenta (9, 10). A small number of clinical studies demonstrate a correlation between low estradiol levels and adverse pregnancy outcomes. In 1992, Check et al. demonstrated that serum estradiol levels were significantly lower during pregnancies that ended in miscarriage (11). This observation was confirmed again in 2022 when Deng et al. reported that levels of estradiol during pregnancy were significantly lower in miscarried pregnancies compared to normal pregnancies (12). Recent reports demonstrate that low estradiol levels are correlated with adverse pregnancy outcome, like preeclampsia (13–15) and small for gestational age babies (16, 17). It is not clear whether these low estradiol levels are a cause or consequence of the pregnancy complications; however, estradiol biosynthesis tended to be lower at a gestational age preceding preeclampsia (18).

Estradiol production takes place via conversion of cholesterol into pregnenolone, dehydroepiandrosterone (DHEA), androgens and finally estradiol (19). DHEA seems to be a key prohormone for estradiol production, as estradiol levels in postmenopausal women increase with DHEA supplementation (20). DHEA supplementation prior to pregnancy has been suggested to increase the rate of spontaneous pregnancies in women with diminished ovarian function (21). Furthermore, Tagawa et al. found a physiologic increase of blood DHEA levels in the first and second trimester of pregnancy (22). However, the impact of DHEA supplementation on estradiol levels during pregnancy is currently unknown.

In the NeoFertility clinic, Dublin, Ireland, treatment of early pregnancy is aimed to reduce the risk of miscarriage for women with a history of infertility and/or recurrent miscarriage. Over the past 12 years, evaluation and treatment in early pregnancy has included tracking serum estradiol levels. For women with low estradiol levels, supplementing with estradiol (in the earlier years of this cohort) or DHEA (in the later years of this cohort) has been used to attempt to normalize serum estradiol levels and reduce the risk of miscarriage. These treatments were part of a multifactorial approach to restore reproductive function based on symptoms and serial measurement of hormone levels, which evolved during the study period. To get more insight into estradiol levels throughout pregnancies resulting in miscarriage or term births, and to assess the potential impact of estradiol or DHEA supplementation in patients with low serum estradiol in pregnancy, we retrospectively analysed serum estradiol levels and pregnancy outcome in pregnant patients who were or were not treated with estradiol or DHEA.

## Materials and methods

### Setting

Between 2009 and 2017, a restorative reproductive medicine (RRM) clinic (now known as NeoFertility) operated in two sites in Ireland, Galway, and Dublin. All patients are medically managed for underlying health issues and treated while attempting to conceive naturally. There were five physicians providing services during this time, all of whom were licensed for family medicine in Ireland. All had received training in RRM, specifically natural procreative technology, which uses the Creighton Model fertility chart to assess menstrual cycle and reproductive function (23, 24). They underwent mentoring with the clinic medical director (PB) to RRM approaches and clinic procedures.

### Medical records and data collection

The data used for this study were routinely collected from patients during their initial and subsequent visits as recorded in the medical records. Additional clinical information customarily collected through phone, mail, e-mail, or survey, and placed in the chart. Data required for this study were abstracted from the existing medical records and entered into a database, with secondary verification of the information occurring as needed. Extracted data were anonymized with unique identifiers.

### Routine checking of estradiol levels during pregnancy

Starting in 2009, the clinic routinely checked serum estradiol levels repeatedly in all pregnancies. Blood draw for serum estradiol analysis was performed in the morning. The analyses were performed by clinical laboratories throughout Ireland and reported in standard units of pmol/L. The precise day of gestation at each blood draw was determined, as described below. Levels were checked weekly in very early pregnancy, prior to an ultrasound to establish early pregnancy viability, which was usually performed around 7 weeks gestation. After the pregnancy was noted to be viable on ultrasound, estradiol levels continued to be checked at intervals throughout pregnancy.

### Reference data for estradiol in pregnancy

**Figure 1:**
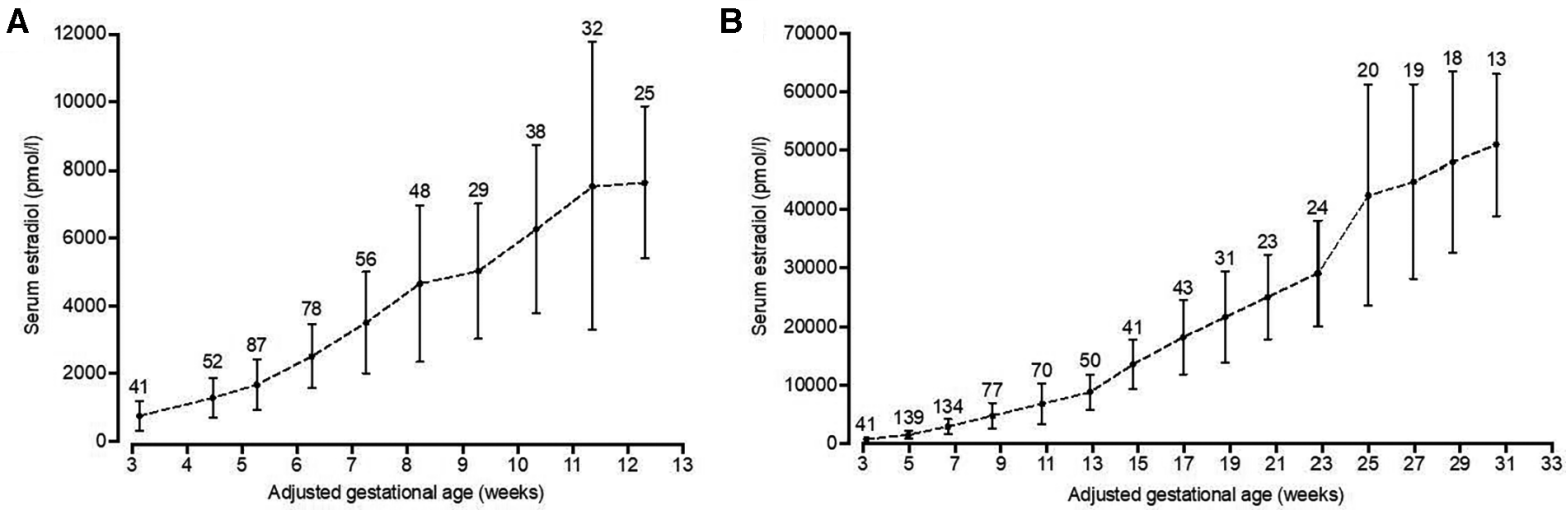
Serum estradiol levels throughout pregnancy. Serum estradiol levels of patients with the last menstrual period between April 2012 and July 2017 (*n* =  l04), who had term deliveries (≥ 37 weeks) and were not supplemented with estradiol, DHEA or pregnenolone, were retrospectively analyzed. Patients with multiple pregnancies or diagnosed with preexisting or gestational diabetes mellitus were excluded from analysis. Pregnancies were quasi-randomly selected within this time frame with the aim to include at least 100 pregnancies. For the first trimester, estradiol levels were averaged from weekly intervals (**A**), while for the full pregnancy biweekly intervals were used (**B**). Precisetime points from weekly and biweekly intervals were determined by averaging the days of estradiol analysis within these intervals. Data are presented as mean with standard deviation. The number above the line indicates the number of measurements per time point.

To identify patients with low estradiol levels in early pregnancy, starting in 2009, a reference range for levels of estradiol in pregnancies that proceeded to term was used from prior work by Check and colleagues, as shown in (Table 1) (11). Additionally, for this current paper, we generated a reference range of estradiol in this clinic population by analyzing retrospectively estradiol levels from 104 pregnancies of women who had term live singleton births, who were not supplemented with estradiol, DHEA or pregnenolone, and who did not have gestational diabetes mellitus. For the first trimester of pregnancy, the estradiol level was analyzed in one week intervals: from 3 weeks 0 days to 3 weeks 6 days, from 4 weeks 0 days to 4 weeks 6 days, and so on. Also, for the entire pregnancy, the estradiol level was analyzed in 2-week intervals: From 3 weeks 0 days to 4 weeks 6 days, from 5 weeks 0 days to 6 weeks 6 days, and so on. Each estradiol level was assigned to its associated weekly or biweekly interval and all estradiol levels of all patients for each time interval were averaged as well as the precise day of gestation within each time interval. This resulted in a clinic-specific reference range for estradiol values in pregnancies, shown in Figure 1, which we used for all subsequent analyses in this paper. In this group, we also explored whether estradiol levels varied by maternal age.

**Table 1:** Threshold of serum estradiol used to initiate oral supplementation of estradiol or DHEA and desired target serum levels^a^.

Adjusted gestational age (weeks)	Threshold E2 concentration (pmol/L)	Normal concentration (pmol/L)	Miscarriage concentration (pmol/L)
4	500	778	642
5	700	1,053	715
6	1,000	1,545	693
7	1,500	2,154	510
8	2,000	2,693	1,141

^a^ Adapted from Check JH, Lurie D, Davies E, Vetter B. Comparison of first trimester serum estradiol levels in aborters versus nonaborters during maintenance of normal progesterone levels. *Gynecol Obstet Invest* 1992;34(4):206–10.

### Analysis of relative estradiol levels during pregnancy

Relative serum estradiol levels were obtained by dividing the absolute estradiol value for each patient and gestational age to the respective reference level of the associated gestational age interval in the clinic-specific reference data (reference levels shown in Figure 1), yielding a percentage of the gestational-age specific estradiol level. The relative serum estradiol levels before vs. after estradiol or DHEA supplementation were obtained by calculating the average of all relative estradiol levels obtained 1–10 days before vs. the average of all available relative estradiol levels obtained 1–6 weeks after supplementation, for each pregnancy.

### Patient inclusion and exclusion criteria

**Figure 2:**
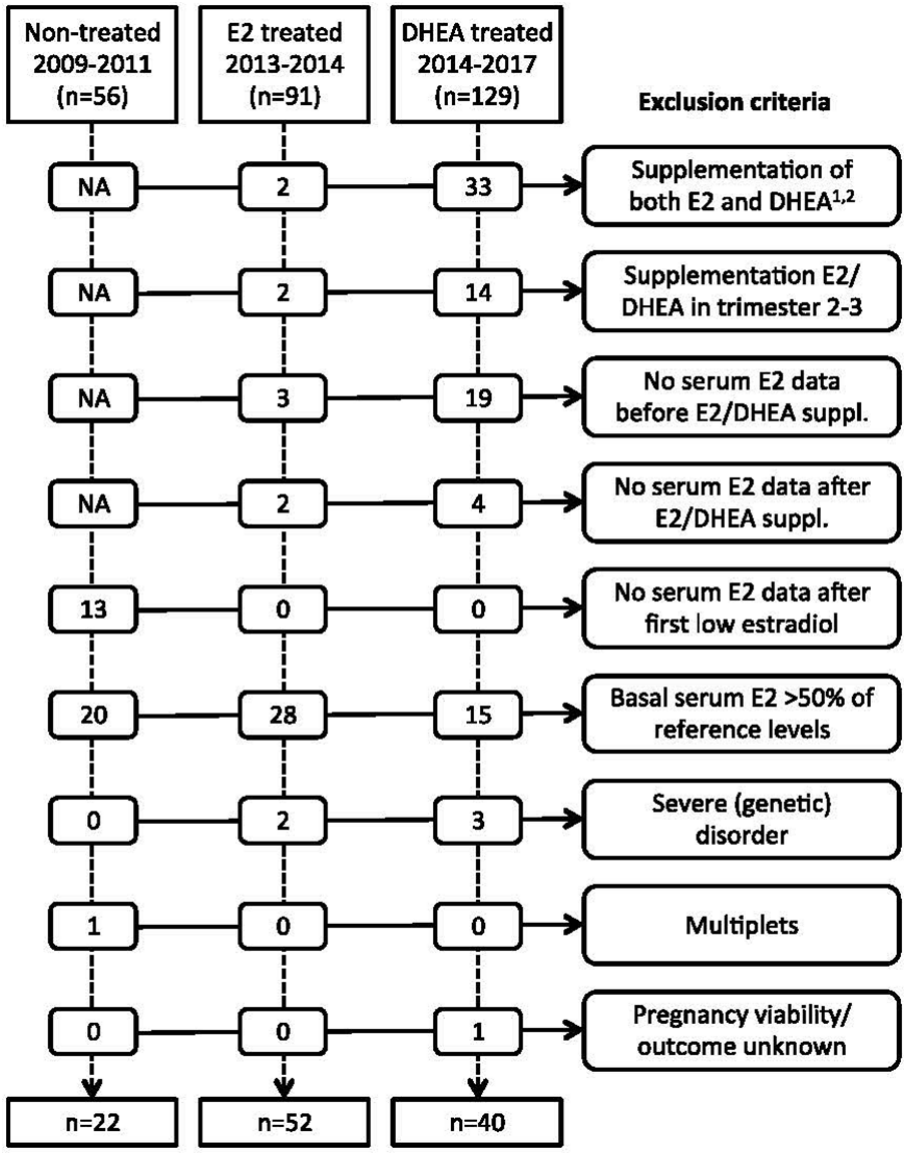
Overview of exclusion criteria. NA, not applicable. ^1^For the year 2017, patients with both estradiol and DHEA treatment were not extracted from patient database. Consequently, the 129 DHEA treated patients and 33 excluded patients for combined treatment from 2014- 2017 represent an underestimation. ^2^There were no patients who received pregnenolone before estradiol or DHEA supplementation or within the first 6 weeks of estradiol or DHEA supplementation. E2, estradiol; DHEA, dehydroepiandrosterone.

The inclusion criteria for the main analysis were all pregnancies clinically identified with low estradiol levels in the first trimester of pregnancy, who either did not receive supplementation with estradiol or DHEA (2009–2011), or who were treated with estradiol (2013–2015), or DHEA (2015–2017). Exclusion criteria were having a basal relative serum estradiol >50% of the reference level (specific for gestational age), multiple gestation, receiving supplementation with both estradiol and DHEA, having received supplementation only in the second or third trimester, or not having serum estradiol levels available before or after supplementation was started, and presence of a severe genetic disorder of the fetus. The number of women excluded for each criterion is given in Figure 2.

### Supplementation with estradiol or DHEA

Estradiol and DHEA are available by prescription only in Ireland. Supplementation was by prescription, with the medication obtained from a pharmacy.

### Identification of gestational age, miscarriage, viable pregnancies and small/large for gestational age babies

Gestational age was determined clinically by estimated day of ovulation (peak mucus day) (25, 26), and confirmed by early pregnancy ultrasound with crown-rump length, usually at 7 weeks gestation. Miscarriage was defined as loss of pregnancy before 24 weeks of gestational age. The presence of small or large for gestational age at birth was determined by using the 10th percentile values from the Intergrowth-21st standards for newborn weight (27). We assigned the birth weight at a gestational age of × weeks and 0–3 days to week x, while days 4–6 were assigned to week x + 1.

### Statistical analysis

Data are presented as mean with standard error of the mean (SEM), unless indicated otherwise. One-way ANOVA with Bonferroni correction was applied to evaluate serum estradiol levels between estradiol and DHEA supplemented patients before or after supplementation. One-way ANOVA with Tukey's Honestly Significant Difference (HSD) test was applied to evaluate pregnancy outcome parameters. Logistic regression was used to determine the odds of miscarriage for patients given estradiol, or DHEA, as compared to neither hormone, unadjusted, and adjusted for woman's age, history of prior miscarriage, and history of prior live birth, which are characteristics associated with the risk of miscarriage. We also adjusted for the prescribed use of other medications, besides estradiol or DHEA, which had substantial differences in prevalence between the treatment groups.

### Ethics approval

The study was approved by Beacon Hospital Research Ethics Committee (BHREC), reference number BEA0187. The analysis was conducted under additional ethics approval from the University of Utah, IRB #80947. Data were abstracted from usual clinical data sources and patient anonymity was maintained by recording data as de-identified prior to analysis. There was no requirement for written informed consent of participants.

## Results

### Patients

For the main analysis, we analyzed pregnancies identified with baseline estradiol levels <=50% of the gestational age reference range, who received no estradiol or DHEA treatment (*n* = 22; 2009–2011), or who received estradiol (*n* = 52; 2013–2015), or who received DHEA (*n* = 40; 2015–2017). The clinical characteristics of these pregnancies (114 pregnancies from 110 women) are given in Table 2. The mean woman's age was in the range 36–37 years; over 80% had prior pregnancies, and the mean number of miscarriages among those with prior pregnancies was 2.1–2.4. Most women (86%–92%) received progesterone during pregnancy, as well as other treatments shown.

**Table 2:** Characteristics and other treatments of 114 pregnant patients with low serum estradiol in early pregnancy by treatment.

	Treatment		
	No Estradiol or DHEA 2009–2011	Estradiol 2013–2015	DHEA 2015–2017
Medication dose [mg; mean (range)]	—	4.0 [2.0–6.0]	24.2 [10.0–50.0]
Total patients	22	52	40
Mean age	37.3	36.1	37.2
Previous live birth (%)	50.0	50.0	55.0
Conceived always miscarried (%)	40.9	28.8	37.5
Mean number of previous miscarriage	2.1	2.1	2.4
Never previously conceived (%)	13.6	21.2	10.0
Other treatments during current pregnancy (%)^a^			
Vitamin B6	9.5	7.7	10.0
Vitamin B12	9.5	9.6	5.0
Selenium	—	11.5	27.5
Aspirin^b^	19.0	28.8	37.5
Enoxaparin	4.8	7.7	5.0
Progesterone	85.7	92.3	90.0
Levothyroxine^c^	14.3	19.2	40.0
Hydrocortisone	4.8	1.9	-
Metformin	9.5	9.6	5.0
Low-dose Naltrexone^d^	38.1	40.4	75.0
Prednisolone^e^	4.8	46.2	60.0
Pregnenolone^f^	—	—	20.0

DHEA = dehydroepiandrosterone.

^a^ Folic acid, Omega-3 fish oil and vitamin D3 were routinely prescribed to all patients.

^b^ Aspirin 75 mg given for abnormal thrombophilia screen.

^c^ Levothyroxine for borderline thyroid dysfunction to keep TSH levels between 1–2 iu/L.

^d^ Low dose Naltrexone 3–4.5 mg nightly for persistent fatigue, low mood, anxiety, PMS and dysmenorrhea.

^e^ Prednisolone 5 mg once per morning for PCOS, hyperandrogenemia or low DHEA.

^f^ Started after first 6 weeks of DHEA supplementation.

### Serum estradiol levels in pregnancies proceeding to term

**Figure 3:**
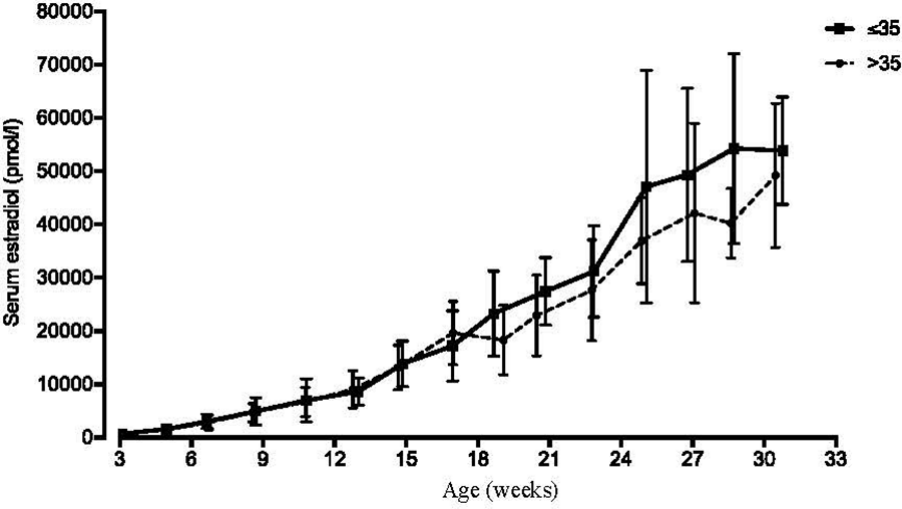
Serum estradiol levels in younger vs older pregnant woman. Serum estradiol levels of patients not supplemented with estradiol from 2012 to 2017 (*n *= 104), who had term deliveries, were retrospectively analyzed. (See Figure 1 and Table 3.) For this figure, patients were stratified in 35 years (*n* = 51, average of 32.5 years) and >35 years (*n *= 53, average of 38.2 years) at the day of conception. Precise time points from weekly and biweekly intervals were determined by averaging the days of estradiol analysis within these intervals. Data are presented as mean with one standard deviation.

As described above, reference data for serum estradiol in pregnancy were generated from patients with full term pregnancies from 2012 to 2017, who did not receive estradiol or other medication that might increase estradiol levels, including DHEA and pregnenolone. With a goal of analyzing about 100 pregnancies, 126 pregnancies were selected. After excluding multiple pregnancies or those with preexisting or gestational diabetes mellitus, 104 out of 126 pregnancies were included. A complete overview of clinical characteristics and treatments for this reference population is presented in Table 3. Mean serum estradiol at one week after estimated ovulation (p7) was 745 pmol/L (*n* = 41) (Figure 1A). Throughout the first trimester these estradiol levels steadily increased, leading to a mean 7,643 pmol/L (*n* = 25) at the 12th week of pregnancy. In the second and third trimester, serum estradiol levels kept increasing until the 31st week to mean 50,986 pmol/L (*n* = 13), after which the number of data points was considered insufficient to calculate mean estimates (Figure 1B). In addition, to explore whether age affected serum estradiol levels in this patient population, the 104 patients were split in patients of ≤35 years (*n* = 51, average of 32,5 years) and >35 years (*n* = 53, average of 38,2 years) at the day of conception. Estradiol levels were not statistically different between these age groups, although they tended to be lower in the elder group after 19 weeks of pregnancy (Figure 3).

**Table 3:** Characteristics of reference population: patients with normal serum estradiol in early pregnancy and subsequent term pregnancies; data from these women were used to derive the referent values in Figure 1.

All patients (*n*)	104
Age (mean)	35.4
Previous live birth (percent yes)	43.4
Conceived always miscarried (percent yes)	21
Never conceived (percent yes)	30
Previous number of miscarriage in woman who conceived (mean)	1.2
Treatments during pregnancy^a^	
• Vitamin B6 (%)	—
• Vitamin B12 (%)	1.9
• Selenium (%)	10.6
• Aspirin (%)	26.0
• Enoxaparin (%)	1.0
• Progesterone (%)	85.6
• Levothyroxine (%)	25.0
• Hydrocortisone (%)	—
• Metformin (%)	18.3
• Low-dose Naltrexone (%)	48.1
• Prednisolone (%)	18.3

^a^ Folic acid, Omega-3 fish oil and vitamin D3 were standardly prescribed to all patients during pregnancy.

### DHEA supplementation is effective to increase serum estradiol

From 2011 onwards, pregnant woman with low blood estradiol levels were identified clinically based on thresholds which were obtained from Check et al. (11) (Table 1). Women with low estradiol were supplemented with oral estradiol in 2013–2015 and in 2015–2017 with oral DHEA. After having established an internal reference estradiol curve for full-term pregnancies as described above and shown in Figure 1, cohorts of estradiol- and DHEA-supplemented patients were established, based on inclusion and exclusion criteria noted above, with exclusion criteria presented in Figure 2. This led to the inclusion of 52 out of 91 estradiol-supplemented patients (last menstrual period between May 2013 and January 2015) and 40 out of 129 DHEA-supplemented patients (last menstrual period between April 2015 and September 2017).

**Figure 4:**
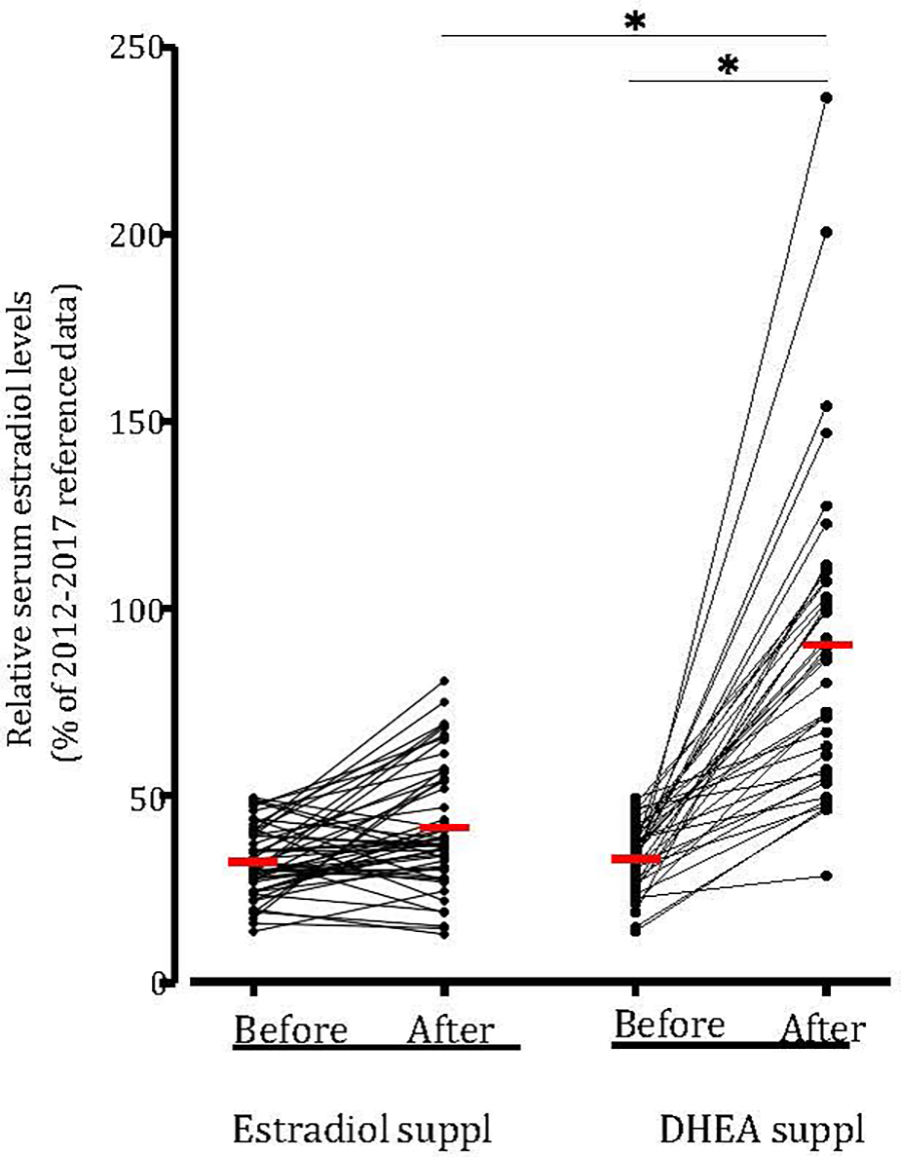
DHEA supplementation increased relative serum estradiol levels in all patients. Serum estradiol levels (in pmol/L) of all patit:nts with low estradiol kvds and suppkmt:nkd with estradiol (*n* = 52) or with DHEA (*n* = 40) compared to 2012–2017 reference data and the relative serum estradiol levels 1–10 days before and 1–6 weeks after supplementation . Values were averaged and compared (red hash mark). *p < 0.05 statistically significant difference. Suppl, supplementation.

**Figure 5:**
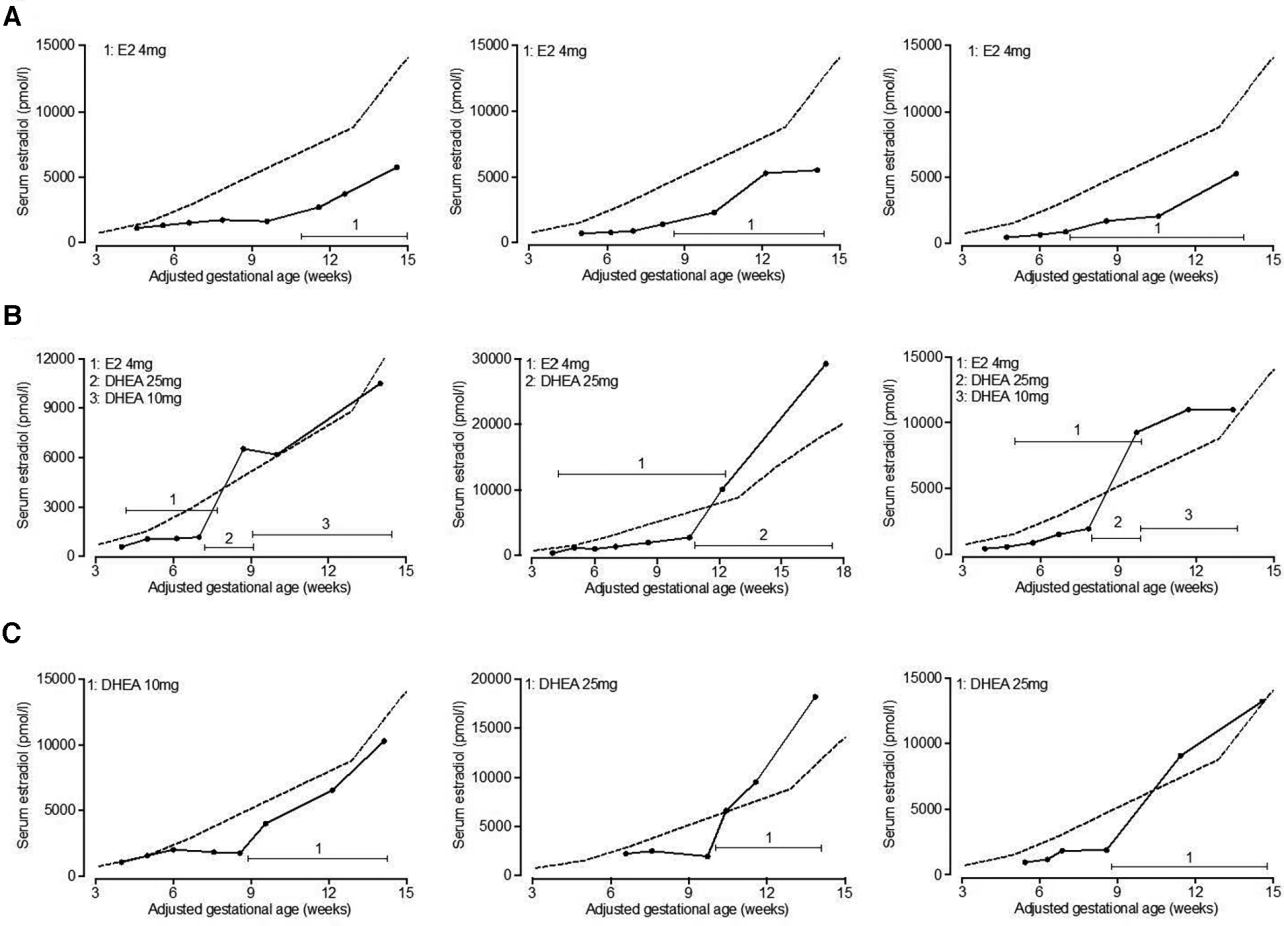
Serum estradiol levels in representative patients supplemented with estradiol or DHEA. Serum estradiol levels in patients supplemented with estradiol or DHEA were retrospectively analyzed. Serum estradiol levels of representative patients are shown who initially exhibited low serum estradiol in relation to 2012–2017 reference levels (indicated by dashed line) and were subsequently treated with estradiol only (**A**), estradiol followed by DHEA (**B**) or DHEA only (**C**). Total daily dose of estradiol and DHEA is indicated. E2, estradiol; DHEA, dehydroepiandrosterone.

On average, both estradiol and DHEA supplementation started at 6.9 weeks of gestation. Baseline relative estradiol levels were a relative mean of 32.3% and 33.2% of reference levels for the estradiol and DHEA group respectively, which was not significantly different. After supplementation for 1–6 weeks, this reached a mean of 41.6% (*p* = 0.14, compared to baseline level) and 90.2% (*p* < 0.0001, compared to baseline level) (median of 37.7% and 87.2%). DHEA supplementation led to significantly higher relative serum estradiol levels as compared to estradiol supplementation (*p* < 0.0001). Oral estradiol did not increase serum estradiol levels to 100% of reference level in any patient, while 35% of DHEA supplemented patients reached this level (Figure 4). This response to DHEA supplementation is illustrated by representative patients treated with estradiol or DHEA only and in patients who were first treated with estradiol, followed by DHEA (Figure 5). In 17 patients with estradiol supplementation there was a decrease in relative serum estradiol levels, while all patients supplemented with DHEA had an increase.

### Estradiol and DHEA supplementation associated with lower incidence of miscarriage as compared to non-treated patients

**Figure 6:**
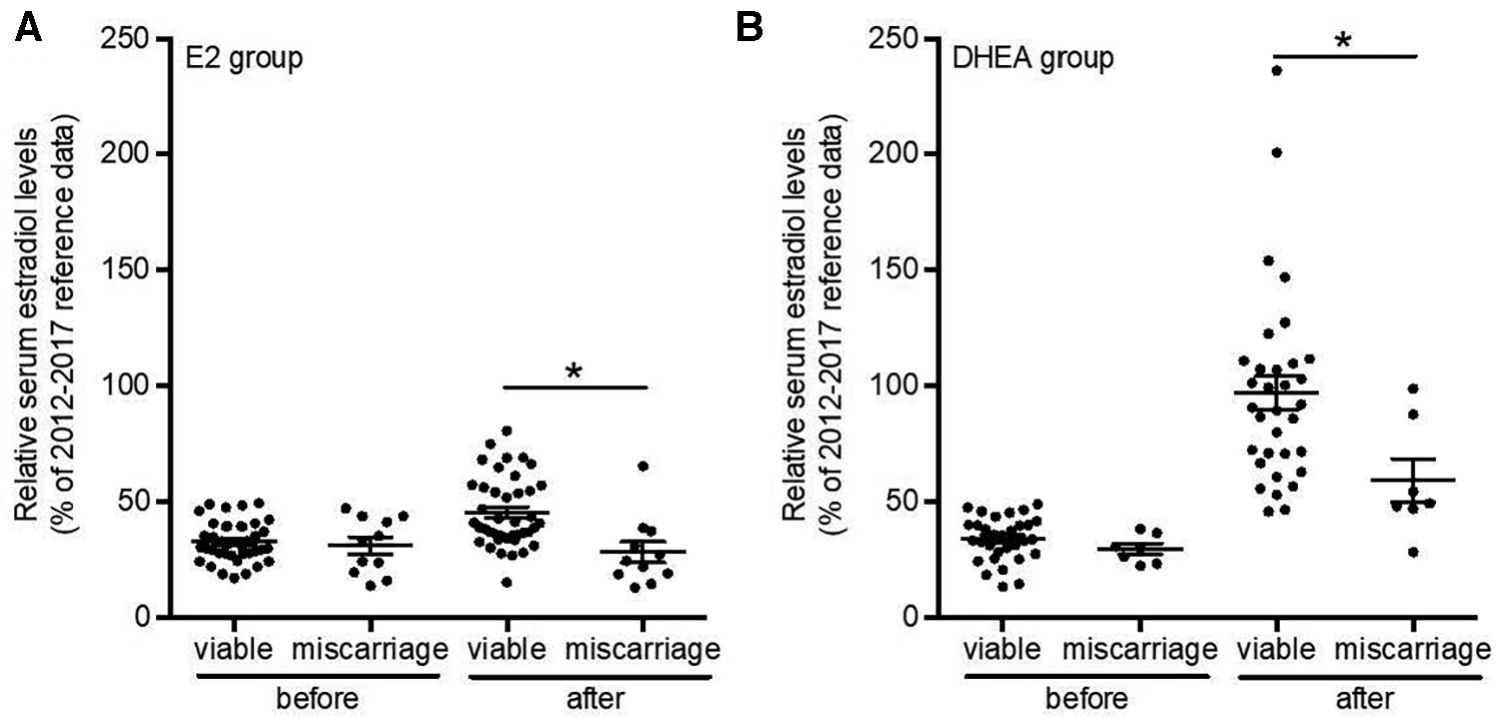
Relative serum estradiol levels after estradiol or DHEA supplementation are highest in viable pregnancies. Serum estradiol levels of all patients with low serum estradiol levels and supplemented with estradiol (*n* = 52) or with DHEA (*n* = 40) with known birth outcome were analyzed and relative serum estradiol levels 1-10 days before and 1–6 weeks after supplementation and compared between viable pregnancies and those leading to miscarriage. *p < 0.05. Suppl, supplementation; E2, estradiol; DHEA, dehydroepiandrosterone.

To determine the potential effect of increasing serum estradiol on the rate of pregnancy loss, pregnancy outcomes in patients supplemented with estradiol (2013–2015 cohort) or DHEA (2015–2017 cohort) were compared with untreated patients (2009–2011 cohort); all patients initially had low estradiol levels in early pregnancy. The patient characteristics and other treatments were presented in Table 2. As shown in Table 4, the latter group the miscarriage rate was 45.5%, while this was 17.5% for the patients supplemented with DHEA (*p* = 0.038) and 21.2% (*p* = 0.067) for those supplemented with estradiol. There was no significant difference in the rates of low birth weight or pre-term deliveries among these groups, although the numbers of these events were small. Comparing serum estradiol levels in patients with subsequent viable pregnancy or miscarriage we found that in both estradiol and DHEA supplemented patients, pregnancies resulting in miscarriage had a blunted increase of serum estradiol with supplementation (Figure 6). Finally, we also compared the start and dose of estradiol and DHEA supplementation in the viable pregnancies and the miscarriages. We found that the average start of estradiol and DHEA supplementation was respectively 7.3 and 7.0 weeks in the viable pregnancies and 5.6 and 6.4 weeks with miscarriage while the average maximal dose was respectively 4.1 and 24.1 mg in the viable pregnancies and 4.4 and 26.4 mg with miscarriage, indicating that the full-term pregnancies with low serum estradiol were not related to a higher dose or earlier administration.

**Table 4:** Estradiol or DHEA supplementation and pregnancy outcome in 114 pregnant women with low serum estradiol levels in early pregnancy.

	Treatment		
	No Estradiol or DHEA 2009–2011	Estradiol 2013–2015	DHEA 2015–2017
Total patients	22	52	40
Pregnancy outcomes *n* (%)			
Miscarriage	10 (45.5)	11 (21.2)	7 (17.5)*
Premature delivery	0 (0)	4 (7.7)	3 (7.5)
Term delivery	11 (50.0)	36 (69.2)	30 (75.0)
Missing	1 (4.5)	1 (1.9)	0 (0)
Births	11	40	33
Fetal outcomes *n* (%)			
Very low birth weight (<1,500 gr)	0	0	0
Low birth weight (1,500–2,500 gr)	1 (9.1)	0	2 (6.1)
Preterm (<37 weeks	0	4 (10.0)	3 (9.1)
Small for gestational age	2 (18.2)	3 (7.5)	2 (6.1)
Large for gestational age	4 (36.4)	6 (15.0)	6 (18.2)

DHEA = dehydroepiandrosterone.

*p < 0.05 as compared to “No Estradiol or DHEA”.

In a logistic regression model, the odds ratio (unadjusted) for miscarriage was 0.32 for treatment with estradiol, and 0.25 for treatment with DHEA. In models adjusted for woman's age, prior miscarriage, prior live birth, and/or the use of naltrexone and prednisolone, the adjusted odds ratios were very similar, but not statistically significant when naltrexone and prednisolone use was added to the adjustment (Table 5).

**Table 5:** Odds ratio of miscarriage with estradiol or DHEA treatment, in reference to no treatment, in women with low serum estradiol levels in early pregnancy.

Number of Pregnancies	Treatment		
	No Treatment 22	Estradiol 52	DHEA 40
	Odds ratio, (95% confidence interval)^a^		
Unadjusted	1 reference	**0.32 (0.11, 0.94)**	**0.25 (0.08, 0.82)**
Model 1: adjusted for woman's age, prior miscarriage, prior live birth	1 reference	0.33 (0.08, 1.28)	**0.19 (0.04, 0.81)**
Model 2: adjusted for use of naltrexone, and use of prednisolone.	1 reference	**0.26 (0.08, 0.86)**	0.27 (0.07, 1.03)
Model 3: adjusted for woman's age, prior miscarriage, prior live birth, use of naltrexone, and use of prednisolone.	1 reference	0.32 (0.07, 1.39)	0.26 (0.05, 1.34)

^a^ Odds ratios that are statistically significant are bolded.

### No reports of abnormalities in sexual development with long-term follow-up of children from DHEA-treated patients

A clinical follow-up was obtained 5–7 years after birth, using the same questionnaire for all patients who received DHEA during pregnancy. The questionnaire informed for birth parameters, mode of delivery, complications for fetus or mother during pregnancy or at time of birth, concerns for motor or cognitive development of the child and for any health-related issues of children. The questionnaire was completed for 29 out of 33 children. In around 20% of pregnancies complications were experienced during pregnancy and at birth (Table 6). Few concerns were reported regarding the child's cognitive or motor development, while in 34.5% of children health-related concerns were reported including asthma (*n* = 3), progressive hearing loss (*n* = 1), Blount's disease (*n* = 1), migraine (*n* = 1), hydronephrosis (*n* = 1) and enlarged tonsils (*n* = 1). There were no reports of any sexual abnormalities.

**Table 6:** Maternally reported clinical outcomes from DHEA-treated pregnancies, 5–7 years after birth.

	*N* (%)
Total number of pregnancies with maternal report	29 (100)
Pregnancies with complications in mother	1 (3.4)
Complications in mother	
Hypermesis	1 (3.4)
Pregnancies with complications in fetus	6 (20.7)
Complications in fetus	
Placenta praevia	1 (3.4)
Preeclampsja	1 (3.4)
Growth restriction	1 (3.4)
Gestational diabetes	3 (10.3)
Bleeding	2 (6.9)
Mode of delivery	
Vaginal	18 (62.1)
C-section planned	8 (27.6)
C-section unplanned	3 (10.3)
Pregnancies with complications at time of birth	6 (20.7)
Complications at time of birth	
C-section unplanned	3 (10.3)
Born premature	2 (6.9)
Trouble breathing after birth	1 (3.4)
Children with concerns about motor development	2 (6.9)
Children with concerns about cognitive development	1 (3.4)
Children with other health-related issues	10 (34.5)
Child health-related issues	
(Suspected) asthma	3 (10.3)
Progressive hearing loss	1 (3.4)
Blount's disease	1 (3.4)
Cow milk allergy	1 (3.4)
Hemangioma lip	1 (3.4)
Occupational therapy observation	3 (10.3)
Migraine	1 (3.4)
Hip dysplasia	1 (3.4)
Hydronephrosis	1 (3.4)
Large tonsils	1 (3.4)

## Discussion

The essential role of estradiol in pregnancy has been known for many decades; however, the impact of low estradiol levels in pregnancy outcome has had relatively little investigation. In this retrospective analysis in pregnancies of women who attended the NeoFertility clinic, Dublin for subfertility or pregnancy complications in 2009–2017, we found that DHEA supplementation was very effective in increasing low serum estradiol levels, more so than direct estradiol supplementation. We also found that the percentage of miscarriages in the DHEA supplemented groups was significantly lower as compared to similar patients who did not receive DHEA or estradiol. No apparent difference in percentage of babies with low birth weight, preterm deliveries or small/large for gestational age babies were found. A clinical follow-up questionnaire to mothers about the children of the DHEA supplemented pregnancies did not reveal any apparent increase in health concerns or sexual morphology, although the number of children studied is small.

### Miscarriage coincides with low estradiol levels

Miscarriage is estimated to occur in up to 30% of pregnancies, depending on the population, with about half of these occurring within the first weeks after conception (28, 29). Although genetic aberrations are thought to cause around 50% of miscarriages in the first trimester (30, 31), the underlying cause in the majority of miscarriages is mostly not identified (32). It is critical to further identify the pathophysiological mechanisms to ultimately provide treatment, especially for women with recurrent miscarriage. In 1991, Check et al. demonstrated a strong correlation between pregnancy outcome and serum estradiol levels (11). Low estradiol would strongly indicate a deficiency in steroid production due to a lack of LH stimulation, or cellular response to LH during early pregnancy. It could also indicate a deficiency of the fetal adrenal-placenta unit during and after the transition to placental production of estradiol at 8–10 weeks. Supplementation of progesterone, is a more common method of treating presumptive corpus luteum deficiency in early pregnancy to reduce the risk of miscarriage (33) and 86%–92% of the patients in this study were supplemented, with no differences between the three treatment groups (Table 2).

### Serum estradiol levels in healthy full-term pregnancies

Several older studies demonstrated a rise in serum estradiol after conception followed by further increases until delivery (11, 34, 35). These studies used radioimmunoassay to determine estradiol, while currently serum estradiol is mostly measured by non-radioisotope labelled immunoassays or liquid chromatography/tandem mass spectrometry-based methods (36, 37, 38). The latter methods have also been used to determine serum estradiol in healthy pregnancies (39, 40). The estradiol assays in this study were conducted at different clinical laboratories across Ireland, introducing an unknown level of between-lab variation. Thus, the reference levels used for this analysis cannot be directly compared to other laboratories and settings.

### DHEA supplementation rapidly increases serum estradiol levels

Among women with low serum estradiol levels in early pregnancy, the mean relative serum estradiol level increased from 33.2% to 90.2% (*p* < 0.0001), after supplementation with DHEA.

### Association of supplementation with reduced number of miscarriages

Among women with low serum estradiol in early pregnancy and no supplementation, the incidence of miscarriage was 45.5%, as compared to 21.2%, and 17.5% for patients supplemented with estradiol and DHEA, respectively. In multivariable models, adjusting for the use of low dose naltrexone and prednisolone (other medications that differed between the treatment groups), and/or for women's age, prior miscarriage, and prior live birth, there was a consistent reduced odds (0.19–0.33) of miscarriage with the supplementation of DHEA or estradiol, although only some of the models had effect estimates that were statistically significant. There might be an improved outcome when supplementation is started very early in pregnancy, or prior to pregnancy. A previous study demonstrated that prolonged DHEA supplementation before pregnancy increased the number of spontaneous pregnancies in couples who were preparing for IVF (21). Another prior study across two centers found that women with diminished ovarian reserve who were supplemented with DHEA for up to two months prior to IVF treatment had an unexpectedly low rate of miscarriage (about 15%) (41).

### Possible mechanisms of action

Together with progesterone, estradiol is essential for remodeling of the uterus to facilitate embryo implantation and placentation, also known as decidualization (42). Furthermore, estradiol stimulates the proliferative phase of endometrium, has vasodilation effects and is important for angiogenesis and therefore neovascularization (43–45), which is crucial to ensure transport of oxygen and nutrients to the growing fetus.

It is believed that DHEA primarily functions as a steroid precursor for the production of androgens and estrogens (20). During the luteal phase of the cycle and the first 10 weeks of pregnancy, most of the progesterone and estradiol are produced by the corpus luteum (46). Therefore, DHEA supplementation is most likely being utilized by the luteinized theca and/or granulosa cells for this steroid production. We cannot rule out peripheral and or adrenal utilization however, especially when ovarian function is deficient, as is for example the case in post-menopausal women (20). After 10 weeks of gestation, it is the placenta that utilizes DHEA to make estrogen, as discussed below.

### Safety concerns

A potential risk of estradiol supplementation is the potential development of thrombosis (47, 48), which could also negatively affect placental development (49, 50). This concern coupled with the fact that estradiol supplementation was less effective leads to a preference for the use of DHEA during pregnancy.

There is no literature available on prolonged DHEA supplementation in pregnancy. Nonetheless, there are various studies regarding long-term DHEA supplementation prior to IVF to improve pregnancy outcome (21, 51). In most of these studies 25 milligrams of DHEA was taken three times daily by mouth for up to six months (51–53). DHEA supplementation was associated with a decreased number of miscarriages (51, 53). Serum DHEA or estradiol levels were not measured in any of these studies (51). The side effects with these dosages were minimal; however, those reported, like hair loss, oily skin and acne vulgaris, are related to its androgenic effects (51). In postmenopausal women oral supplementation of 50 mg DHEA per day for 52 weeks was found to be safe (54). There are no published data regarding prolonged DHEA supplementation during pregnancy.

At the NeoFertility clinic serum estradiol was carefully monitored during pregnancy and DHEA was only supplemented with low serum estradiol levels. As DHEA supplementation in this study was restricted to woman with low serum estradiol levels and never exceeded 50 mg/day, we believe that abnormally high serum androgen levels and severe side effects are unlikely. However, androgen levels were not measured in this study. An increase of androgen blood levels could impact fetal development, particular for female fetuses.

The luteal to placenta shift in synthesis of estradiol is completed around 8–10 weeks of gestation (9, 10, 55). However, the placenta can only make estrogen if there are sufficient levels of DHEA for enzymatic conversion. The DHEA for estrogen production comes from a combination of maternal and fetal sources, with the fetal adrenal gland becoming predominant around 10 weeks of gestation (55). Furthermore, the production of DHEA by the fetal adrenal gland is tightly regulated (55, 56), therefore it is very likely that an increased maternal source of DHEA would reduce production by the fetal adrenal gland, preventing excess androgenization. In our long-term survey on children from DHEA-supplemented pregnancies, no sexual abnormalities were reported. In future studies, measurement of fetal androgens and the anogenital distance at birth would be valuable for addressing this potential concern.

Any drug or supplement used during pregnancy should be highly scrutinized to prevent harm to the developing fetus. An infamous example of this is the use of Diethylstilbestrol (DES) for the prevention of miscarriage and premature delivery from 1938 to 1971 (57). Diethylstilbestrol was also a form of estrogen supplementation, but it is a synthetic nonsteroidal estrogen which has a modified chemical structure and 3–4 times higher affinity for the estrogen receptor (57). This difference is likely the reason it was classified as carcinogenic to humans in 2000 (57) and is no longer in use. This is contrasted by DHEA which is bio-identical, itself considered a weak hormone and a physiologic requirement during pregnancy which is tightly regulated.

### Study limitations

Our observations of the link between DHEA supplementation and estradiol levels were made in a clinical setting and were not found in previously published literature. A major limitation of this study, however, is its retrospective nature, and relatively small number of pregnancies treated with DHEA. The assignment of treatment was not randomized but was changed over time periods of the clinical operations based on clinical data and symptomatology. Although the treatment groups had similar clinical characteristics, the potential for other unmeasured confounding factors exist. Other temporal trends in treatment (as noted in Table 2) may have contributed to differences in outcome for the patients who were treated with DHEA. Randomized, controlled studies are needed to confirm our observations and to further assess for adverse effects. All patients in this study were being evaluated and treated for subfertility and/or history of pregnancy loss andthese results may not necessarily apply to other types of patients.

## Conclusions

In conclusion, our results support prior work that low serum estradiol in early pregnancy is associated with miscarriage, demonstrate that DHEA supplementation increases serum estradiol levels in women who have low estradiol levels during early pregnancy, and suggest that administering DHEA has the potential to reduce the miscarriage rate in pregnancies with low serum estradiol levels. While reducing miscarriage is the purpose of our treatment, the multi-factorial nature of miscarriage makes it difficult to measure definitively an association with one treatment or biomarker in a clinical setting. However, low estradiol levels are an easily measurable biomarker that can be potentially treated, and should be further validated as a routine test and target of therapy for recurrent miscarriage.

## Data Availability

The datasets presented in this article are not readily available because data were assembled from private medical records and are not publicly available. Data will be made available to the editors of the journal or query if necessary upon request. Requests to access the datasets should be directed to Phil Boyle phil.boyle@neofertility.ie. Data regarding any of the subjects in the study has not been previously published unless specified. Data will be made available to the editors of the journal for review or query upon request.
